# SARS-CoV-2 does not replicate in embryonated hen’s eggs or in MDCK cell lines

**DOI:** 10.2807/1560-7917.ES.2020.25.25.2001122

**Published:** 2020-06-25

**Authors:** Ian G Barr, Cleve Rynehart, Paul Whitney, Julian Druce

**Affiliations:** 1 The WHO Collaborating Centre for Reference and Research on Influenza, The Peter Doherty Institute for Infection and Immunity, Melbourne, VIC, Australia; 2 Department of Microbiology and Immunology, The University of Melbourne, The Peter Doherty Institute for Infection and Immunity, Melbourne, VIC, Australia; 3 Faculty of Science and Technology, Federation University, Gippsland, VIC, Australia; 4 Victorian Infectious Diseases Reference Laboratory, The Peter Doherty Institute for Infection and Immunity, Melbourne, VIC, Australia

**Keywords:** Influenza, SARS-CoV-2, vaccine, respiratory, air-borne infections, viral infections, influenza, severe acute respiratory syndrome – SARS, influenza virus, biosafety, vaccines and immunisation, laboratory

## Abstract

The advent of COVID-19, has posed a risk that human respiratory samples containing human influenza viruses may also contain SARS-CoV-2. This potential risk may lead to SARS-CoV-2 contaminating conventional influenza vaccine production platforms as respiratory samples are used to directly inoculate embryonated hen’s eggs and continuous cell lines that are used to isolate and produce influenza vaccines. We investigated the ability of these substrates to propagate SARS-CoV-2 and found that neither could support SARS-CoV-2 replication.

The majority of the global production of influenza vaccine is currently performed in embryonated hen’s eggs [1] with a growing proportion in mammalian cell lines such as Madin–Darby canine kidney cells (MDCK) [2]. Both of these methods derive their initial viruses from clinical respiratory samples of patients with influenza-like illness (ILI), which are inoculated directly into these substrates [3]. A small proportion of these influenza-positive samples may now be co-infected with severe acute respiratory syndrome coronavirus 2 (SARS-CoV-2) and influenza virus, as reported in a recent study by Kim et al. 2020 [4]. They found that 0.9% of samples positive for SARS-CoV-2, from individuals in Northern California, were also positive for influenza virus (with another 5.2% of SARS-CoV-2 cases co-infected with respiratory syncytial virus (RSV), and 15.5% with another respiratory pathogen) in a period towards the end of the influenza season (3–25 March 2020) in the United States (US) [4]. During the peak of future influenza seasons, the rate of SARS-CoV-2/influenza virus co-infections may increase if, as might be expected, SARS-CoV-2 co-circulates with influenza, thereby enhancing the risk of contamination of influenza vaccine seed stocks. Careful screening using sensitive molecular-based methods should reduce the possibility of this occurring, however, there still exists a theoretical risk of viable virus being present, which may compromise candidate influenza vaccine viruses and increase the risk to workers in the influenza vaccine industry and laboratory scientists, who routinely work under a biosafety level (BSL)2 containment and not the higher levels (BSL3 or BSL4) required to amplify SARS-CoV-2 safely.

We investigated if SARS-CoV-2 could be propagated in embryonated hen’s eggs or the most used mammalian cell lines that are currently used for propagating influenza viruses (in both the diagnostic laboratory and in vaccine production), MDCK cells and variants of this cell line.

## Inoculating and passaging SARS-CoV-2 and influenza viruses on influenza virus substrates

Briefly we inoculated two different levels (100 median tissue culture infectious dose (TCID_50_) and 1,000 TCID_50_) of Vero (African green monkey kidney cells; ATCC CCL-81) cell-grown SARS-CoV-2 in 100 µL of phosphate buffered saline (PBS) containing 1.45 mg/mL of neomycin sulphate (Pharmacia & Upjohn, Peapack, New Jersey, US) and 0.25 mg/mL of polymyxin B sulphate (Xellia Pharmaceuticals, Copenhagen, Denmark) into the amniotic cavity or the allantoic cavity of three 15-days-old or three 11-days-old, embryonated hen’s eggs (vaccine certified) respectively and incubated them for 3 days at 35 °C. Fluids were harvested from the eggs and pooled according to their inoculum dose and site, then re-passaged a further two times by re-inoculating 100 µL of a 1:2 dilution of fluid in PBS and antibiotics into the same sites into three eggs/site/inoculum dose and harvesting fluids (but not pooling) after 3 days at 35 °C. This process mimics influenza virus isolation from clinical samples (amniotic inoculation) and virus growth in vaccine production (allantoic inoculation) using the embryonated hen’s egg platform. As a control, embryonated hen’s eggs from the same batch of eggs were separately inoculated allantoically with a 1×10^6^ dilution of a previous egg passage of B/Victoria/2110/2019 or B/Victoria/2113/2019 (both B/Victoria lineage viruses) for a single passage.

For attempted propagation in mammalian cells, 5×10^4^, 5×10^3^ or 5×10^2^ TCID_50_ inoculums of SARS-CoV-2 derived from infection of Vero cells were applied in Dulbecco’s Modified Eagle Minimum Essential media (DMEM) to T25 flasks (Corning cell culture flasks, surface area 25 cm^2^, canted neck, cap (vented); Sigma-Aldrich, Sydney, Australia) containing confluent cell monolayers of either regular MDCK cells (ATCC CCL-34), MDCK-SIAT-1 [5], MDCK-TMPRSS2 [6], MDCK-hCK [7] or Vero cells and after 60 min the inoculum was removed and media containing 4 µg/mL trypsin (Sigma-Aldrich) was added and cells were incubated at 37 °C for 6 days (as previously described [8]). Subsequently 100 µL samples from this first passage were inoculated onto fresh cell monolayers in T25 flasks as detailed above and incubated at 35 °C for a further 6 days. As a control, another set of flasks containing these same cell lines were separately inoculated with human influenza virus isolates (A/Victoria/13/2020 (H1N1pdm09) or B/Victoria/2117/2019 (B/Victoria-lineage)) with 200 µL of a 1×10^3^ dilution of an existing isolate, using the same method as above, for a single passage.

## Assessing SARS-CoV-2 and influenza viruses’ propagation on influenza virus substrates

Samples from the egg-passaged and mammalian cell-passaged SARS-CoV-2 samples were examined for propagation of SARS-CoV-2, visually inspected for embryo viability (eggs), for cytopathogenic effects – CPE (cells), and by real-time RT-PCR (both cells and eggs) targeting the RNA-dependent RNA polymerase (RdRp) gene of SARS-CoV-2 with cycle thresholds (Ct) rounded up or down to the nearest integer. Briefly, for cell samples, 200 µL were subjected to RNA extraction using the QIAmp 96 Virus QIAcube HT kit (Qiagen, Hilden, Germany) and the RNA was eluted in 60 µL, while for egg samples, RNA was extracted from 140 µL with QIAamp mini-Viral RNA columns (Qiagen) and eluted in 60 µL. Reverse transcription and real-time PCR were performed as previously described [8] using an Applied Biosystems Fast Real Time PCR machine (Applied Biosystems, Foster City, California, US). Influenza RNA was detected as previously described [9].

## Ethical statement

All experiments with SARS-CoV-2 were conducted in BSL-3 facilities and the use of embryonated hen’s eggs was approved by the University of Melbourne Animal Ethics Committee (2015136.1).

## Effects of inoculating SARS-CoV-2 and influenza viruses on hen’s eggs and Vero cells

Table 1 shows the results from the egg-inoculations. The initial inoculum of SARS-CoV-2 had Ct values of 28 for 100 TCID_50_ and 25 1,000 TCID_50_. Following the first amniotic passage into three individual eggs, the pooled amniotic fluids had a Ct of 38 for the 100 TCID_50_ inoculum and 36 for the 1,000 TCID_50_ inoculum, and allantoically inoculated eggs gave Cts of 37 and 33 respectively for allantoic fluids. These Ct values continued to increase to undetectable levels (Ct > 40) upon further passaging for both sites of inoculation. In contrast when the 100 TCID_50_ and the 1,000 TCID_50_ were inoculated into Vero cells after 3 days at 37 °C they gave reduced Ct values of 17 and 15 respectively, indicating a high level of replication had occurred. No effect on embryo viability was detected following inoculation with SARS-CoV-2. Eggs inoculated separately with two influenza B viruses (initial influenza B Cts of 26 and 27) both replicated to high levels as indicated by the highly reduced influenza B Cts of 11 (for B/Victoria/2110/2019) and 10 (for B/Victoria/2113/2019), after a single passage (Table 1). Eggs inoculated with buffer alone had no detectable SARS-CoV-2 RdRp signal.

**Table 1:** Quantification of SARS-CoV-2^a^ and influenza^b^ viruses’ RNA after passages into embryonated hen’s eggs and Vero cells

Virus	Route of inoculation	SARS-CoV-2 TCID_50_ (Ct value)	Passage 1 Ct value	Passage 2 Ct value	Passage 3 Ct value
**SARS-CoV-2**	Amniotic				
	E1	100 (28)	38^c^	ND	ND
	E2	100 (28)		ND	ND
	E3	100 (28)		ND	ND
	E1	1,000 (25)	36^c^	ND	ND
	E2	1,000 (25)		ND	ND
	E3	1,000 (25)		ND	ND
	Allantoic				
	E1	100 (28)	37^c^	ND	ND
	E2	100 (28)		ND	ND
	E3	100 (28)		ND	ND
	E1	1,000 (25)	33^c^	ND	ND
	E2	1,000 (25)		ND	ND
	E3	1,000 (25)		ND	ND
	Vero cells				
	VC	100 (28)	17	NA	NA
	VC	1,000 (25)	15	NA	NA
**Influenza**	Allantoic				
• B/Victoria/2110/2019	E	NA (26)^d^	11	NA	NA
• B/Victoria/2113/2019	E	NA (27)^d^	10	NA	NA

Ct: cycle threshold; E: egg; NA: not applicable/not done; ND: not detected; SARS-CoV-2: severe acute respiratory syndrome coronavirus 2; TCID_50_: median tissue culture infectious dose; VC: Vero cell.

^a^ Quantification of SARS-CoV-2 RNA after each passage in eggs and Vero cells was assessed with a real-time RT-PCR assay targeting the RNA-dependent RNA polymerase (RdRp) gene.

^b^ Quantification of influenza virus RNA after one egg passage was assessed with a previously described real-time RT-PCR assay [9].

^c^ At passage 1, E1, E2,E3 harvests were pooled and then inoculated into three separate eggs at passage 2 then respectively carried onto passage 3.

^d^ Allantoic isolate initially diluted 10 ^− 6^.

## Effects of inoculating SARS-CoV-2 and influenza viruses on MDCK cell lines and Vero cells

The results testing SARS-CoV-2 propagation in mammalian cell lines are shown in Table 2. At the first passage there was residual SARS-CoV-2 detected in most MDCK cell lines (Ct values ranged from not detected down to 25, with no CPE) while the Vero cells showed lower Cts of 15, 15, 14 in T25 culture flasks (and extensive CPE) when inoculated with 5×10^4^, 5×10^3^, 5×10^2^ TCID_50_ respectively, 6 days after inoculation, indicating high levels of SARS-CoV-2 replication. When a 100 µL sample of each MDCK cell line from passage 1 was applied to new cells (of the same type), this second passage showed no CPE or decrease in Ct values, producing signal levels equivalent to background (Cts: 37–39), indicating that no SARS-CoV-2 replication had occurred. In contrast when 100 µL from passage 1 in Vero cells (Day 6; originally inoculated with 5×10^2^ TCID_50_ SARS-CoV-2) was applied to fresh Vero cells, this resulted in a Ct of 12, again indicating high levels of replication at this second passage (Table 2). 

**Table 2:** Quantification of SARS-CoV-2^a^ RNA after passages into various MDCK cell lines and Vero cells

Cell line	Passage 1		Passage 2
	SARS-CoV-2 inoculum TCID_50_	Flask 1; flask 2 Ct values	Pooled flasks 1 + 2 Ct value
MDCK	5×10^4^	27; 26	37
	5×10^3^	29; 29	39
MDCK-SIAT-1	5×10^4^	26; 25	37
	5×10^3^	29; 28	37
MDCK-TMPRSS2	5×10^4^	26; 27	37
	5×10^3^	30; 30	37
MDCK-hCK	5×10^4^	ND; 26	37
	5×10^3^	28; 35	37
Vero	5×10^4^	14	NA
	5×10^3^	15	NA
	5×10^2^	15	12

Ct: cycle threshold; MDCK: Madin–Darby canine kidney cells; NA: not applicable/not done; ND: not detected; SARS-CoV-2: severe acute respiratory syndrome coronavirus 2; TCID_50_: median tissue culture infectious dose.

^a^ Quantification of SARS-CoV-2 RNA after each passage in MDCK cell lines and Vero cells was assessed with a real-time RT-PCR assay targeting the RNA-dependent RNA polymerase (RdRp) gene.

As a control, MDCK cell lines inoculated separately with either an influenza A or B virus (initial influenza A and B Cts of 25 and 29 respectively) both replicated to high levels as indicted by the highly reduced Cts (influenza A Cts of 9–12 and influenza B Cts of 11–14 respectively) after a single passage, however, Vero cells showed little signs of influenza replication (Table 3). No CPE was detected in any of the MDCK cell lines inoculated with SARS-CoV-2. Uninfected cell lines all showed no detectable SARS-CoV-2 RdRp signal or CPE.

**Table 3:** Quantification of influenza^a^ viruses’ RNA after passage into various MDCK cell lines^b^ and Vero cells

Cell line	Virus	Passage 1 Ct value
MDCK	A/Victoria/13/2020^c^	10
	B/Victoria/2117/2019^d^	12
MDCK-SIAT-1	A/Victoria/13/2020^c^	9
	B/Victoria/2117/2019^d^	11
MDCK-TMPRSS2	A/Victoria/13/2020^c^	12
	B/Victoria/2117/2019^d^	14
MDCK-hCK	A/Victoria/13/2020^c^	11
	B/Victoria/2117/2019^d^	13
Vero	A/Victoria/13/2020^c^	29
	B/Victoria/2117/2019^d^	36

Ct: cycle threshold; MDCK: Madin–Darby canine kidney cells.

^a^ Quantification of influenza virus RNA after one passage in cells was assessed with a previously described real-time RT-PCR assay [9].

^b^ Same cell lines as in Table 2

^c^ An A(H1N1)pdm09 virus – initial inoculum diluted 1×10 ^− 3^; Ct = 25.

^d^ A B/Victoria-lineage virus – initial inoculum diluted 1×10 ^− 3^; Ct = 29.

## Discussion

These results are in line with a previous report [10] that showed that the closely related SARS-CoV-1 (previously known as SARS or SARS-CoV) did not replicate in 9- or 13-days-old embryonated chicken eggs or 17-days-old embryonated turkey eggs when inoculated allantoically. Furthermore, SARS-CoV-1 given by an intra-tracheal challenge to a range of avian species (including geese, ducks, chickens, turkeys and quails), showed only residual levels of RT-PCR virus specific product. There was no infectious virus isolated from inoculated birds or any specific histological lesions from a range of tissues examined and plasma samples showed no seroconversion to SARS-CoV-1, indicating that this virus did not infect these species [9]. In studies on permissiveness of coronavirus to standard MDCK cells, Kaye et al. 2006 [11] showed that SARS-CoV-1 did not replicate as measured by CPE, indirect fluorescence antibody (IDFA) or quantitative RT-PCR, but did replicate in a wide range of mammalian continuous cell lines (derived from kidney epithelium, kidney fibroblasts and liver cells) including Vero/VeroE6 cells. This was supported by a study of the lack of growth of a seasonal coronavirus (E229) in the MDCK-33016 cells developed for cell-based influenza vaccine production [12]. Even when MDCK cells were engineered to increase the infectivity or yield for influenza viruses (SIAT-1, TMPRSS2, hCK), no replication of SARS-CoV-2 was observed in this present study, unlike when transmembrane serine protease 2 (TMPRSS2) was expressed in VeroE6 cells in another study, which resulted in enhanced replication of SARS-CoV-2 compared with VeroE6 or standard Vero cells [13]. All of the MDCK cell lines tested in the present study, efficiently propagated influenza A and influenza B isolates, and embryonated hen’s eggs efficiently propagated influenza B viruses when inoculated allantoically.

## Conclusions

The studies described above give us confidence that even if a clinical sample, containing both human influenza and SARS-CoV-2, was inoculated into substrates used to prepare seeds for influenza vaccine production (embryonated chicken eggs or MDCK-based cell lines), SARS-CoV-2 would be unlikely to be propagated and would be undetectable after a small number of passages. This finding will reassure influenza vaccine production staff and laboratory scientists who might be concerned about potential exposure to SARS-CoV-2 and also suggests that loss of potentially important influenza candidate vaccine viruses or final vaccine lots due to SARS-CoV-2 contamination is unlikely.
